# Supplementary material for: The Fusion Gene BPI-LY, Encoding Human Bactericidal/Permeability-Increasing Protein Core Fragments and Lysozyme, Enhanced the Resistance of Transgenic Tomato Plants to Bacterial Wilt
Source: Plants (Basel). 2025 Jun 20;14(13):1897. doi: 10.3390/plants14131897 (PMC12251675; doi:10.3390/plants14131897)

Supporting Information Figures legends

Figure S1 Western blot of target protein expression levels

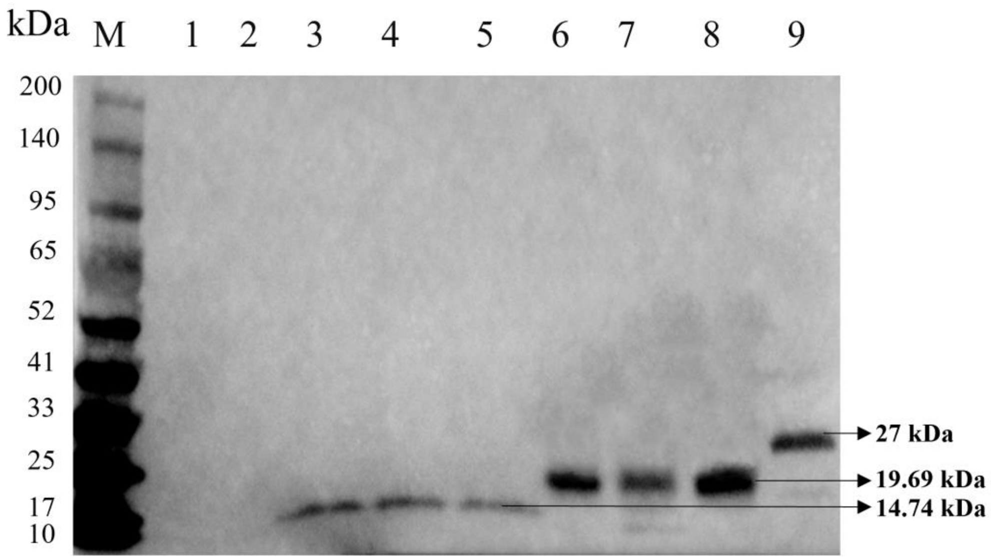

Note: M: protein Maker. 1: GS115 yeast expression supernatant of 120 h. 2: empty vector GS115-pPIC9K yeast expression supernatant of 120 h. 3-5: LY yeast expression supernatant of 72 h, 96 h, 120 h. 6-8: BPI-LY yeast expression supernatant of 72 h, 96 h, 120 h. 9: positive control (eGFP protein-27 kDa (Yeasen, Shanghai, China)).

Protein supernatant samples were separated on a 150 g/L SDS-PAGE (Beyotime, Shanghai, China) gel and subsequently transferred onto a polyvinylidene difluoride (PVDF (Beyotime, Shanghai, China)) membrane via electrotransfer. The membrane was blocked overnight at 4 °C using blocking buffer containing 5% skim milk (Beyotime, Shanghai, China) powder. It was then incubated with a 1:1000 dilution of mouse anti-His antibody (Beyotime, Shanghai, China) at room temperature for 1 h, followed by three washes with Tris-buffered saline (TBS (Beyotime, Shanghai, China)). Next, the membrane was incubated with a 1:1000 dilution of goat anti-mouse IgG antibody (Beyotime, Shanghai, China) conjugated with horseradish peroxidase (HRP) at room temperature for 1 h. After additional washing steps, the membrane was treated with enhanced chemiluminescence (ECL (Beyotime, Shanghai, China)) reagent for 1 min, and the results were visualized using a chemiluminescence imager (Clinx, Shanghai, China).

Figure S2 Phenotypic observations of plant populations at 7 days after inoculation

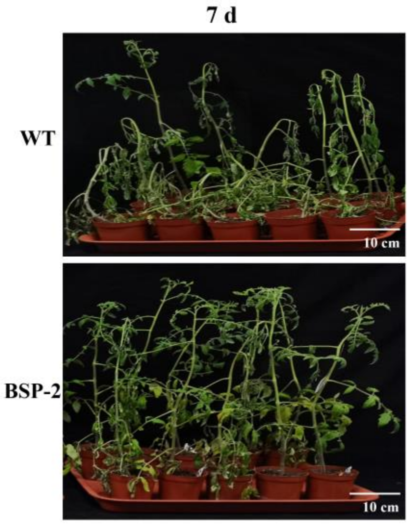

Note: WT: wild type; BSP-2: T_2 homozygous offspring of the transgenic tomato plants; 7 d: inoculation for 7 days.

Figure S3 *sp-BPL-LY* sequence in the pVCT2455-*sp-BPL-LY* vector

>*sp-BPL-LY* in pVCT2455 (654 bp)

atgggatttgtgctcttttcacaattgccttcatttctcctcgtctctacactcctcttgttcctcgtgatctcccactcttgccgtgcccagaacatcaa
gatcagtgggaaatggaaggcacagaagcgtttcctcaagggatctggcaagagcaaagtcgggtggctgatccaactcttccacaagaag
ggtggcggtgggtcaggtggaggtggctctatggctaaggtgttcgagcgttgtgagttggctcgtaccttgaagcgtttgggaatggacggtt
atcgtggtatctctttggctaactggatgtgtttggctaagtgggaatctggttacaacactcgtgctaccaactacaacgctggagaccgttcaa
ctgactacggtatcttccagatcaattctcgttactggtgtaacgacggtaagaccccaggtgccgttaacgcttgtcacttgtcttgttctgctttgt
tgcaggacaacatcgctgacgctgttgcttgtgctaaacgtgtggttcgtgacccacaaggtatccgtgcttgggttgcttggcgtaatcgttgc
caaaaccgtgacgtgcgtcaatacgttcagggttgtggtgtggctagctacccatacgatgtgcctgattacgcttaa//

The base sequence of *sp-BPI-LY* was adjusted according to the codon preference of tomatoes, and the artificial synthesis was entrusted to Wuhan Jinkairui Bioengineering Co., Ltd. (Wuhan, China, https://www.genecreate.cn/(accessed on 18 April 2025)). The *BPI-LY* gene was obtained by PCR amplification from the *sp-BPI-LY* sequence.

The deduced amino acid sequence of *sp-BPI-LY* (24.05 KDa)

MGFVLFSQLPSFLLVSTLLLFLVISHSCRAQNIKISGKWKAQKRFLKgsgKSKVGWLIQLFH
KKggggsggggsMAKVFERCELARTLKRLGMDGYRGISLANWMCLAKWESGYNTRATNYN
AGDRSTDYGIFQINSRYWCNDGKTPGAVNACHLSCSALLQDNIADAVACAKRVVRDPQGI
RAWVAWRNRCQNRDVRQYVQGCGVASYPYDVPDYA

(1) "MGFVLFSQLPSFLLVSTLLLFLVISHSCRAQN" is a secretory signal peptide of the tobacco PR1 gene (X06361). After secretion to the outside of the cell, it will be cut between the "QN", and the signal peptide will be removed;

(2) "NIKISGKWKAQKRFLK": A segment (amino acids 84-99) of the bactericidal/permeability-increasing protein BPI (human Bactericidal Permeability Increasing

Protein CAA48684) can bind to the lipopolysaccharides of gram-negative bacteria, thereby increasing their membrane permeability and killing them (Kim H, Jang JH, Kim SC, Cho JH. Enhancement of the antimicrobial activity and selectivity of GNU7 against Gram-negative bacteria by fusion with LPS-targeting peptide. Peptides. 2016 Aug;82:60-66. doi: 10.1016/j.peptides.2016.05.010), (To develop Gram-negative selective AMPs that can inhibit the effects of lipopolysaccharide (LPS)-induced sepsis, we added various rationally designed LPS-targeting peptides [amino acids 28-34 of lactoferrin (Lf28-34), amino acids 84-99 of bactericidal/permeability];

(3) "KSKVGWLIQLFHKK": Another fragment (amino acids 148-161) in BPI (CAA48684) can bind to lipopolysaccharides of gram-negative bacteria and neutralize endotoxins (Chockalingam, A., McKinney, C.E., Rinaldi, M., Zarlenga, D.S. & Bannerman, D.D. A peptide derived from human bactericidal/permeability-increasing protein (BPI) exerts bactericidal activity against Gram-negative bacterial isolates obtained from clinical cases of bovine mastitis. Vet. Microbiol. 2007, 125, 80-90.);

(4) "gsg" and "ggggsggggs": Flexible connecting peptides；the above core fragment of BPI and LY were linked by flexible linker peptides, which ensured that the spatial structure and function of the two were not affected;

(5) "MAKVFERCELARTLKR//NRDVRQYVQGCGV": Human lysozyme（X75362）;

Figure S4 Lysozyme sequence in the GS115 (pPIC9k-*LY*) vector

> *LY* in GS115 (pPIC9k-*LY*) vector (402 bp)

atggctaaggtgttcgagcgttgtgagttggctcgtaccttgaagcgtttgggaatggacggttatcgtggtatctctttggctaactggatgtgttt
ggctaagtgggaatctggttacaacactcgtgctaccaactacaacgctggagaccgttcaactgactacggtatcttccagatcaattctcgtt
actggtgtaacgacggtaagaccccaggtgccgttaacgcttgtcacttgtcttgttctgctttgttgcaggacaacatcgctgacgctgttgcttg
tgctaaacgtgtggttcgtgacccacaaggtatccgtgcttgggttgcttggcgtaatcgttgccaaaaccgtgacgtgcgtcaatacgttcagg
gttgtggtgtggctagc

The deduced amino acid sequence (14.74 kDa)
MAKVFERCELARTLKRLGMDGYRGISLANWMCLAKWESGYNTRATNYNAGDRSTDYGI
FQINSRYWCNDGKTPGAVNACHLSCSALLQDNIADAVACAKRVVRDPQGIRAWVAWRNR
CQNRDVRQYVQGCGVAS

Figure S5 *BPL-LY* sequence in the GS115 (pPIC9k-*BPI-LY*) vector

> *BPL-LY* in GS115 (pPIC9k-*BPI-LY*) vector (537 bp)

atggctaacatcaagatcagtgggaaatggaaggcacagaagcgtttcctcaagggatctggcaagagcaaagtcgggtggctgatccaac

tcttccacaagaagggtggcggtgggtcaggtggaggtggctctatggctaaggtgttcgagcgttgtgagttggctcgtaccttgaagcgttt

gggaatggacggttatcgtggtatctctttggctaactggatgtgtttggctaagtgggaatctggttacaacactcgtgctaccaactacaacgc

tggagaccgttcaactgactacggtatcttccagatcaattctcgttactggtgtaacgacggtaagaccccaggtgccgttaacgcttgtcactt

gtcttgttctgctttgttgcaggacaacatcgctgacgctgttgcttgtgctaaacgtgtggttcgtgacccacaaggtatccgtgcttgggttgctt ggcgtaatcgttgccaaaaccgtgacgtgcgtcaatacgttcagggttgtggtgtggctagc

The deduced amino acid sequence (19.69 kDa)

MANIKISGKWKAQKRFLKGSGKSKVGWLIQLFHKKGGGGSGGGGSMAKVFERCELART LKRLGMDGYRGISLANWMCLAKWESGYNTRATNYNAGDRSTDYGIFQINSRYWCNDGK TPGAVNACHLSCSALLQDNIADAVACAKRVVRDPQGIRAWVAWRNRCQNRDVRQYVQG CGVAS

Figure S6 Structural diagram of the tomato overexpression vector pVCT2455-*sp-BPL-LY*

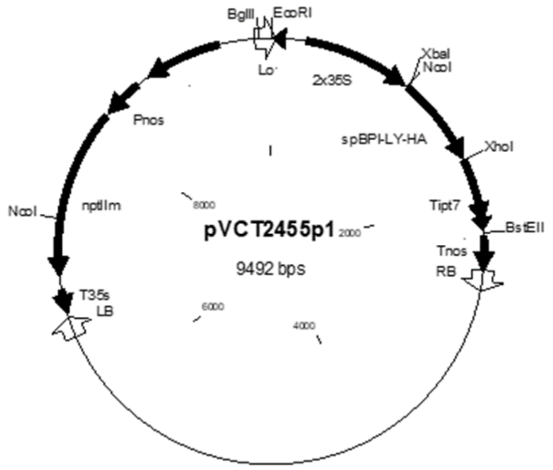

Supplement: Supplementary file 1 [file plants-14-01897-s001.zip › Proofreading=Revised Supplementary Figures.pdf]
