# Supplementary material for: The Fusion Gene BPI-LY, Encoding Human Bactericidal/Permeability-Increasing Protein Core Fragments and Lysozyme, Enhanced the Resistance of Transgenic Tomato Plants to Bacterial Wilt
Source: Plants (Basel). 2025 Jun 20;14(13):1897. doi: 10.3390/plants14131897 (PMC12251675; doi:10.3390/plants14131897)
Supplement: Supplementary file 1 [file plants-14-01897-s001.zip › Proofreading=Revised Supplementary Tables.pdf]

Supplementary Table S1 Transcriptome analysis results of notably differentially expressed genes.

Gene ID	Identifier	log2FoldChange	log2FoldChange	gene description	KEGG	regulation type
		BSP-treatment /BSP-CK	WT-treatment /WT-CK			
gene-GA2ox2	Solyc07g056670.2.1	15.4017343	14.96859433	GA2ox2	K04125 gibberellin 2-oxidase [EC:1.14.11.13] | (RefSeq) GA2ox2, SlGA2ox2; gibberellin 2-oxidase 2 (A)	up
gene-WRKY75	Solyc05g015850.2.1	11.45263416	9.80669294	WRKY75	K18835 WRKY transcription factor 2 | (RefSeq) SlWRKY2; WRKY transcription factor 2 (A)	up
gene-EREB	Solyc09g089930.1.1	8.72139551	4.361545337	EREB	K14516 ethylene-responsive transcription factor 1 | (RefSeq) EREB, TSRF1; ethylene responsive element binding protein (A)	up
gene-SlWRKY33	Solyc09g014990.2.1	8.149243144	8.506983511	protein_coding	K13424 WRKY transcription factor 33 | (RefSeq) SlWRKY33B; WRKY transcription factor 33B (A)	up
gene-SlWRKY41	Solyc01g095630.2.1	7.427066021	9.527445559	WRKY3	K13425 WRKY transcription factor 22 | (RefSeq) WRKY transcription factor 22 (A)	up
gene-LOC101264783	Solyc02g071130.2.1	6.95321106	6.687347241	protein_coding	K13424 WRKY transcription factor 33 | (RefSeq) SlWRKY33A, SlDRW1; WRKY transcription factor 33A (A)	up
gene-SlWRKY33A	Solyc06g066370.2.1	5.640393219	4.661519724	SlWRKY33A	K13424 WRKY transcription factor 33 | (RefSeq) SlWRKY33A, SlDRW1; WRKY transcription factor 33A (A)	up
gene-LOC101262522	Solyc05g051180.1.1	4.280129	4.502709183	protein_coding	K14516 ethylene-responsive transcription factor 1 | (RefSeq) ethylene-responsive transcription factor 1B-like (A)	up

gene-ht2	Solyc09g075820.2.1	4.183228697	3.793022554	ht2	K08150 MFS transporter, SP family, solute carrier family 2 (myo-inositol transporter), member 13 | (RefSeq) inositol transporter 1 (A)	up
gene-LOC543848	Solyc06g076560.1.1	3.416582262	3.008909242	LOC543848	K13993 HSP20 family protein | (RefSeq) type I small heat shock protein 17.6 kDa isoform (A)	up
gene-LOC543600	Solyc10g080770.1.1	3.363967944	3.522107653	LOC543600	K14431 transcription factor TGA | (RefSeq) NIF1; TGACG-sequence-specific DNA-binding protein TGA-2.1 (A)	up
gene-LOC100301944	Solyc06g068460.2.1	3.190569244	4.364839138	LOC100301944	K18835 WRKY transcription factor 2 | (RefSeq) probable WRKY transcription factor 26 (A)	up
gene-PR-1a1	Solyc01g106610.2.1	2.354031973	2.395190611	PR-1a1	K13449 pathogenesis-related protein 1 | (RefSeq) PR-1a1; PR-1a1 protein (A)	up
gene-SlbZIP14	Solyc02g083520.2.1	-5.900890253	-6.797622693	SPGB	K14432 ABA responsive element binding factor | (RefSeq) ABSCISIC ACID-INSENSITIVE 5-like protein 2 (A)	down
gene-SlMYB55	Solyc10g044680.1.1	-4.352533466	-3.509535493	protein_coding	K09422 transcription factor MYB, plant | (RefSeq) transcription factor MYB86-like (A)	down
gene-SlbZIP25	Solyc03g046440.1.1	-3.66033379	-5.874684268	protein_coding	K14432 ABA responsive element binding factor | (RefSeq) protein ABSCISIC ACID-INSENSITIVE 5 (A)	down
gene-LOC101267581	Solyc12g008800.1.1	-3.649328356	-6.922855923	protein_coding	K12133 MYB-related transcription factor LHY | (RefSeq) protein LHY (A)	down
gene-LOC101262486	Solyc11g073120.1.1	-2.89451629	-2.113151851	protein_coding	K09422 transcription factor MYB, plant | (RefSeq) transcription factor MYB48 (A)	down
gene-LOC101261079	Solyc02g036370.2.1	-2.796447247	-2.346805882	protein_coding	K12133 MYB-related transcription factor LHY | (RefSeq) protein LHY (A)	down
gene-LOC101261662	Solyc10g005080.2.1	-2.580258958	-2.58956119	protein_coding	K12133 MYB-related transcription factor LHY | (RefSeq) protein LHY (A)	down

Supplementary Table S2 Primers used in this study.

Primer description	F-primer	R-primer
First nest PCR amplify primers of *LY* in in the pVCT1412 vector	GCTGAAGCTTACGTAGAATTCATGGCTAAGGTGTTCGAG	TTAGTGGTGGTGGTGGTGGTGGCTAGCCACACCACAACCC
Second nest PCR amplify primers of *LY*	GCTGAAGCTTACGTAGAATTCATGGCTAAGGTGTTCGAG	AAGGCGAATTAATTCGCGGCCGCTTAGTGGTGGTGGTGGTG4021
First nest PCR amplify primers of *BPI-LY* in in the pVCT1412 vector	GCTGAAGCTTACGTAGAATTCATGGCTAACATCAAGATC	TCAGTGGTGGTGGTGGTGGTGGCTAGCCACACCACAACCC
Second nest PCR amplify primers of *BPI-LY*	GCTGAAGCTTACGTAGAATTCATGGCTAACATCAAGATC	AAGGCGAATTAATTCGCGGCCGCTCAGTGGTGGTGGTGGTGGT
Detection of the target gene *LY* in the pPIC9k vector and GS115	GCTGAAGCTTACGTAGAATTCATGGCTAAGGTGTTCGAG	GGCAAATGGCATTCTGAC
Detection of the target gene *BPI-LY* in the pPIC9k vector and GS115	GCTGAAGCTTACGTAGAATTCATGGCTAACATCAAGATC	GGCAAATGGCATTCTGAC
Detection of the target gene *sp-BPI-LY* in the *Escherichia coli* vector	ATGACGCACAATCCCACTATCC	CCAGATTCCCACTTAGCCAAACAC
Detection of the target gene *sp-BPI-LY* in the *Agrobacterium tumefaciens* vector	CATTTCTCCTCGTCTCTACACTCCTC	CGTTCGATGACGAAAATGGAAG
Detection of the target gene *sp-BPI-LY* in the transgenic lines	GTGTTCGAGCGTTGTGAGTTGG	CATCGCAAGACCGGCAACAGG
Real-Time PCR primers of *spBPI-LY*	GGAAATGAAGTCTCTCCCACAAAT	TTCGCTAAGAGCCCTGAATATG
Real-Time PCR primers of *SlSOD*	ACTATTACCGACAAGCAGATTCC	CCTCCCTTTCCAAGATCATCAG
Real-Time PCR primers of *SlPOD*	GTGTCAGAAGCCAATGGTGATAT	GGACCAGGTCATTCAGATCAAG
Real-Time PCR primers of *SlPAL*	AGATTGGCCCTTGCATCTATTGG	CAGCTCCCTTGAGTCCATAATC
Real-Time PCR primers of *SlPR5*	TCTCGAGAGAGGTCAGAGTTG	TGCAATCCAGGACTCCACCAC
Real-Time PCR primers of *SlPR10*	GCTGCTGGAGATGGAGGTTGTG	GAGGTACGCTTCGATGGCCTTG
Real-Time PCR primers of *SlPR-NP24*	CACAAGACGATCCTACAAGCAC	CATCAGTACTTGCAGGCATCTC
Real-Time PCR primers of *SlGA2ox2*	GCCATCAAGTTCTTCTCCTCTCC	TGTGTTCGACCCGACCACAATC
Real-Time PCR primers of *SlWRKY33*	ATCACCTGCTTCGCCTTCCTC	CCCTGTAGTTGGCGATGAAAGAG
Real-Time PCR primers of *SlWRKY41*	GCAACACCAAACCATAACGCTG	CGATGTGTAGTGGTGCAGGAG

Real-Time PCR primers of *SlMYB55*	TCCGTACCTGATCCTGATGAC	GGCTGTTGTTGTTGGTTCTG
Real-Time PCR primers of *SlbZIP25*	CTTGAACGAACTATGGGCACAG	TGACGAAGTCCAGCAGTTTC
Real-Time PCR primers of actin gene *SlActin7*	GGTCGGAATGGGACAGAAGGATG	CCTCAGTCAGGAGAACAGGGTG
